# Schistosoma haematobium, *Plasmodium falciparum* infection and anaemia in children in Accra, Ghana

**DOI:** 10.1186/s40794-018-0063-7

**Published:** 2018-04-18

**Authors:** Ruth Nyarko, Kwasi Torpey, Augustine Ankomah

**Affiliations:** School of Public Health, University of Ghana, P. O Box LG 13, Legon, Accra Ghana

**Keywords:** Schistosoma haematobium, *Plasmodium falciparum*, Hemoglobin, Anaemia, School children

## Abstract

**Background:**

Urinary Schistosomiasis and malaria are endemic in Sub-Saharan Africa. There are public health concerns and implications of these parasites. This study sought to assess the prevalence of malaria, urinary schistosomiasis, and anaemia in children of school going age in two municipalities in Ghana.

**Methods:**

A cross-sectional study design was used to investigate the prevalence of *S. haematobium*, *P. falciparum infection* and the haemoglobin concentration of respondents. A total of 404 (231 males and 173 females) school children between ages 9 - 14 years (mean age 11.8 ± 1.4 years) were recruited for the survey. Urine and blood samples were collected using standard operating procedures for urinary schistosomiasis and malaria diagnosis. Haemoglobin concentration was measured using a Hemocue® Hb 201 m.

**Results:**

The prevalence of mono-infection was 4.7 and 12.9% for *S. haematobium* and *P. falciparum* respectively with a small proportion (0.9%) of the respondents infected with both parasites. The prevalence of anaemia in the study population was 59.9%. The risk of developing anaemia was not associated with being infected with any of the parasites. All co-infected children had anaemia.

**Conclusion:**

High prevalence of anaemia was observed within the study population. Prevalence of malaria was higher compared to schistosomiasis. Interventions to address the high levels of anaemia is required within the community.

**Electronic supplementary material:**

The online version of this article (10.1186/s40794-018-0063-7) contains supplementary material, which is available to authorized users.

## Background

Malaria and urinary schistosomiasis are parasitic infections with grave public health implications. Both parasites have been associated with poverty and several related factors which may result in their distribution [1]. Some predisposing factors include low socio economic status, poor sanitation, and limited access to safe water, development of water resource and poor education and awareness play a key role in the transmission and infestation of malaria and schistosome parasites. Children are most vulnerable to schistosome and malaria infection due to their exposure to the infectious agents in activities of daily living.

Urinary schistosomiasis caused by *S. haematobium* is the most common form of schistosomiasis in Ghana. Schistosomes are often referred to as blood flukes because they live in blood vessels. Its transmission requires an intermediate and suitable snail host. Contamination of water bodies with human excreta and urine releases the primary larvae (miracidiae) which infects the snails and develops into free-living secondary larvae in the snail host. Water-related activities such as swimming, fishing, irrigation or farming, fetching of water and washing clothes or dishes expose humans to the cercariae. The cercariae penetrate the skin thereby infecting humans in the process [2, 3]. The creation of dams and urbanization facilitates the development of the habitat for snails and consequently increases the local prevalence of schistosomiasis [2, 3]. *S. haematobium* deposits eggs in the host bladder which induces inflammation and causes ulceration resulting in haematuria [3, 4]. Reported prevalence in Ghana ranged from 1.5 to 52% [5–7].

Malaria in Ghana is commonly caused by the *Plasmodium falciparum* infection which is transmitted through the bite of the female anopheles mosquito [8]. The total parasitaemia prevalence reported among school children in Ashanti Region and Wa West District in Ghana was 42% [9]. The risk of developing low haemoglobin concentration has been associated with *P. falciparum* infection [10]. Malaria parasites break down red blood cells and increase splenic clearance of both infected and non-infected red blood cells [10]. The reported prevalence of coinfection with *P. falciparum* and *S. haematobium* in Africa ranges from 2.84 to 57.1% [10–12].

This study sought to assess the prevalence of malaria, schistosomiasis, and anaemia among children of school-going age in six peri-urban areas located in Ga South and Ga West municipality in Ghana.

## Methods

### Study area

This study was carried out in two different municipalities in Ghana; Ga West and Ga South Municipality. This geographic area was selected due to the presence of water bodies which serves as a source of drinking water for more than 4million people living in the Greater Accra Region. Three schools were selected in each municipality using purposive sampling.

### Study design

An analytical cross-sectional study design was used to investigate the relationship between *S. haematobium*, *P. falciparum* and the haemoglobin concentration of school children. Three schools were selected based on proximity to open source of water in the two districts. Proportionate stratified sampling technique was used to determine the number of participants to be recruited in each grade in all the participating schools. Simple random sampling was used in selecting participants from each grade in the six schools. Children were recruited from grade 3 to grade 6 which constituted an age range of 9 to 14 years.

Sample size determination: a prevalence of 50% (for *S. haematobium and P. falciparum* infection) was used to compute the sample size at a 95% confidence level and a statistical power of 90%. The minimum sample size calculated for this study was 253 participants.

### Data collection and analysis

The study was conducted between April and June 2016. A structured questionnaire was used to collect data from the respondents. Sociodemographic data such as such as name, age, sex, the residence and length of stay in the community. The socio-economic status was measured using a tool developed by the Center for Social Research which has also been used in similar studies (Additional file 1) [13]. The tool combined six variables; type of roofing material, household water source, the occupation of household head, type or availability of toilet facility, floor material and household assets A weight ranging from 0 to 2 was assigned to each asset under the six variables. Weights were assigned based on the value of the assets. The sum of the weights obtained from the six variables was used to derive a score which determined the socioeconomic status for each household. Households were classified as low SES for those with scores less than 4.0, moderate SES for 4.0–6.0 and high SES for a score more than 6.0. However, the tool was modified to be relevant to the context within which the study was carried out.

#### *Plasmodium falciparum* infection

Presence of *P. falciparum* was detected using a Rapid Diagnostic Test Kit (CareStart™, Access Bio, Inc.). The CareStart Kit was used because *P. falciparum* infection is the commonest occurring infection which accounts for more than 90% of all cases of malaria [14]. Each respondent was provided with a clean, dry, screw-capped urine container bearing the same serial number as the questionnaire and instructions were given on the amount of urine to be provided. Urine filtration method was done using Whatman® Nucleopore™ Track-Etched Membranes diameter 25 mm, pore size 12 μm, polycarbonate. The intensity of *S. haematobium* infection (egg count per 10 ml of urine) was determined via microscopy. Light infection was classified as 1-49eggs/10 ml of urine and heavy infection was defined by an egg count equal to or less than 50eggs/10 ml of urine. Haematuria was measured using URIT 10 V urine reagent strips. Haemoglobin (Hb) concentration of respondents was measured using HemoCue Hb 201^+^ and HemoCue Hb 201^+^ Microcuvettes.

Data were entered into Microsoft Excel 2013 and exported into STATA 13. Descriptive statistics were used to analyze the background characteristics of participants and the factors associated with *S. haematobium* and *P. falciparum* infection and coinfection of *S. haematobium* and *P.falciparum*. Differences in the prevalence of mono-infection and coinfection were determined using a chi-square analysis. Multivariate logistic regression analysis was used to predict association with the different infection status as the dependent variable and other independent variables. Differences in the mean hemoglobin level of the different infection status and socio-demographic variables were analyzed using a one-way ANOVA. Values were considered to be significant when *p*-values were less than 0.05.

### Inclusion and exclusion criteria

Children who were not in grade 3 to grade 6 were excluded from the study. Children who did not provide both assent and parental consent were excluded from the study.

## Results

### Background characteristics

The study had a response rate of 97% with 404 out of 414 expected participants responded to the study. More than half (57.2%) of the respondents were males with the majority (63.0%) being between 12 and 14 years old. The children had a mean age of 11.8 ± 1.4 years. Most of the children were from families with moderate socioeconomic status (Table 1).

**Table 1:** Background characteristics of respondents

Background characteristics	Frequency (n)	Percent (%)
Sex		
Male	231	57.2
Female	173	42.8
Age		
9–11	151	37.4
12–14	253	62.6
Grade		
Socio-economic status		
Low	30	7.4
Moderate	227	56.2
High	147	36.4

### S. haematobium, *P. falciparum* and coinfection prevalence pattern

Prevalence of urinary schistosomiasis in the schools ranged from 0 to 10.3%. Out of the 404 respondents, 4.7% (*N* = 19) of the respondents had a positive diagnosis for schistosomiasis based on the egg count by microscopy whilst 5.2% of the participants had haematuria (Table 2). The observed difference in the prevalence of urinary schistosomiasis between the two districts was not statistically significant. Infection with *S. haematobium* was not gender or socioeconomic status dependent. However, there was an increasing trend with increasing age, with the highest prevalence of 6.3% recorded for the age group 12 to 14 years [(OR = 3.87, CI = 1.03–14.41), *P*-value = 0.044] (Table 2). Respondents infected with *S. haematobium* were categorized into the light and heavy infection. Light infection in the study was defined as an egg count of less than 50eggs/10 ml of urine and heavy infection was defined as an egg count of 50 or more eggs per 10 ml of urine. The egg count of respondents ranged from 1 to 204 eggs/10 ml of urine with an average egg count of 2.3 ± 0.9 eggs/10 ml of urine.

**Table 2:** A multivariate analysis showing the prevalence pattern of S. haematobium, *P. falciparum* and coinfection

		*P. falciparum* Infection				S. haematobium Infection				Co-infection
	Number examined	%	OR	95% CI	*p*-value	%	OR	95% CI	*p*-value	%
Total	404	12.9				4.7				1.0
District										
Ga West	208	18.8	1	Ref		4.3	1	Ref		0.9
Ga South	196	6.6	0.30	0.15–0.59	0.001**	5.1	1.02	0.39–2.64	0.06	1.0
Gender										
Female	173	13.4	1	Ref		4.3	1	Ref		1.2
Male	231	12.1	1.02	0.56–1.90	0.925	5.2	1.18	0.45–3.04	0.33	0.9
Age group										
9–11	151	13.9	1	Ref		1.9	1	Ref		–
12–14	253	12.3	0.86	0.46–1.6	0.621	6.3	3.55	1.01–1.49	0.048**	1.6
Socioeconomic status										
Low	80	26.7	1	Ref		3.3	1	Ref		–
Medium	227	10.1	0.28	0.11–0.72	0.009**	6.6	2.34	0.29–18.7	0.423	1.8
High	147	14.3	0.38	0.14–1.00	0.05	3.1	0.67	0.07–6.74	0.734	–

**Significant at the 0.05 probability level

The prevalence of malaria among the study population was 12.9%. Prevalence was higher in Ga West (18.8%) municipality than in Ga South (6.6%) [(OR = 3.87, CI = 0.16–0.59), *P*-value < 0.001]. Prevalence of malaria was neither associated with gender, age or socioeconomic status (Table 2).

Out of the 404 respondents, only 4 (0.9%) school children had concomitant *P. falciparum* and *S. haematobium infection* (Table 2).

The mean haemoglobin level among respondents was 11.6 g/dl ± 1.5 with a range of 5.5 g/dl to 15.1 g/dl. Children within the age group 12–14 years had a higher mean haemoglobin concentration compared to the 9-11 years age group. Grade 6 children had the highest haemoglobin concentration whereas grade 4 respondents had the lowest haemoglobin level. Table 3 shows a significant difference (*p* < 0.05) in the haemoglobin level between *S. haematobium* positive and negative individuals. There was a significant difference in the mean haemoglobin concentration of respondents with light and heavy infection for *S. haematobium*. Children with heavy infection had a lower mean haemoglobin level than children with light infection. *P. falciparum* infected children had lower haemoglobin concentration compared with children not infected with *P. falciparum* (Table 3).

**Table 3:** A Multivariate Analysis of Haemoglobin concentration

	Hemoglobin concentration			
	Hb(g/dl) ± SD	Minimum Hb(g/dl)	Maximum Hb(g/dl)	*P* – value
Total	**11.6 ± 1.5**	**5.5**	**15.1**	
District				
Ga West	11.5 **±** 1.4	5.5	14.5	0.146
Ga South	11.7 **±** 1.5	7	15.1	
Age				
9–11	11.3 **±** 1.5	5.5	14.2	0.001**
12–14	11.8 **±** 1.4	7	15.1	
Sex				
Male	11.6 **±** 1.3	7.7	15.1	0.431
Female	11.5 ± 1.6	5.5	15.1	
Socioeconomic status				
Low	11.4 ± 1.7	5.5	14.1	0.103
Moderate	11.7 ± 1.4	6.2	15.1	
High	11.4 ± 1.5	7	14.7	
Hematuria				
Present	12.3 ± 1.4	10.2	15.1	0.016**
Absent	11.6 ± 1.4	5.5	15.1	
Infection status				
Schistosomiasis Positive	12.3 ± 1.7	8.5	15.1	0.0332**
Schistosomiasis Negative	11.6 ± 1.4	5.5	15.1	
Malaria Positive	11.4 ± 1.2	8.6	13.5	0.3139
Malaria Negative	11.6 ± 1.5	5.5	15.1	
Intensity of S. haematobium infection				
light infection	12.9 ± 1.5	10.7	15.1	0.003**
heavy infection	11.2 ± 1.5	8.5	13.3	

**Significant at the 0.05 probability level

## Discussion

The overall prevalence of 4.7% reported for urinary schistosomiasis in this study was less than the prevalence reported in similar studies in Nigeria and Ethiopia [10, 11, 15]. Previous studies in Galilea, peri-urban community in Ga South municipality and in the Northern region of Ghana respectively reported a prevalence of 52 and 33% for urinary schistosomiasis higher than the prevalence found in this study [6, 7]. The reduced prevalence in this study can be attributed to improvement in sanitation, the supply of safe water, awareness about the disease and the year to year administration of praziquantel to the school children in all the selected schools [15]. The high prevalence of light infection is consistent with similar studies in Nigeria and Malawi [13, 16]. The total prevalence for *P. falciparum* (12.9%), reported in this study was lower than the prevalence in a similar population [9]. The observed difference for *P. falciparum* infection could be attributed to the use of malaria prevention methods such as insecticide-treated nets and insecticides. Previous studies in same study site revealed a prevalence between 54 and 60% for urinary schistosomiasis [17]. The year to year praziquantel chemotherapy could have influenced the reduction in the prevalence of urinary schistosomiasis. Praziquantel has proven effective in reducing the prevalence and intensity of infection and risk of reinfection with S. haematobium [18].

The total prevalence of anaemia in the study was 59.9% which is indicative of a severe public health problem [19]. In Ethiopia, an anaemia prevalence of 81% was reported among children of school going age [15]. Inadequate nutritional intake, low socioeconomic status, and worm infestation are possible risk factors for the observed high prevalence of anaemia in the study.

The mean haemoglobin concentration of the study population was 11.6 ± 1.5 g/dl. This is because the prevalence of mild anaemia was higher than the prevalence of moderate and severe anaemia. This finding is similar to other studies among children of school going age [9, 20].

Although haemoglobin concentration of *P. falciparum* infected children was lower than *S. haematobium* infected respondents, malaria was not a discriminatory factor to anaemia. This could be attributed to the high endemicity of *P. falciparum* among the study respondents resulting in misclassification. Children who were lightly infected with *S. haematobium* had a higher haemoglobin concentration because they are less likely to have haematuria. The *Plasmodium* parasites break down red blood cells and increase the clearance of both infected and uninfected red blood cells and a reduction in erythropoiesis [21]. Similar findings of a higher risk of lower hemoglobin concentration with *P. falciparum* infection was reported in Nigeria [10]. Respondents with light infection had a significantly higher haemoglobin concentration compared with respondents with heavy infection (*p*-value < 0.05) thus contributing to the higher haemoglobin concentration in *S. haematobium* infected respondents. The intensity of *S. haematobium* infection is positively correlated with hemoglobin concentration [15].

All respondents with coinfection had anaemia. In Ethiopia children with concurrent *S. haematobium* and *P. falciparum* infection had a lower hemoglobin concentration than children with mono-infection [15]. The concurrent infection with both parasites may have enhanced their risk of anaemia in the children who were co-infected.

The reported prevalence of hookworm infection in Ghana ranges from 3.2 to 10% [22, 23]. There is currently on-going helminth control program in the two districts. School children are given albendazole treatment for worm infection and praziquantel administration for schistosomiasis. Helminthic infestation particularly hookworms can lead to anaemia. Though the study did not assess the nutritional status and prevalence of helminthic infections, anaemia due to the worm infestation is unlikely given the ongoing interventions in the area.

Further studies, taking into consideration, these confounders should be carried to obtain the true relationship between these risk factors and haemoglobin concentration in school children. This is necessary to understand the causes of the high levels of anaemia for the formulation of appropriate public health intervention.

## Conclusions

High prevalence of anaemia within the study communities is of concern. Investigating common causes of low hemoglobin concentration such as helminth infection and nutritional intake among others is required to address the challenge. Furthermore, public health interventions to reduce the prevalence of malaria and schistosomiasis particularly among school going children is warranted.

## Additional file

Additional file 1: Socioeconomic Status Tool. (DOCX 48 kb)

## Abbreviations

Hb: Hemoglobin
ITN: Insecticide treated net
WHO/ TDR: World Health Organisation/ Tropical Disease Research
