# Supplementary material for: Schistosoma haematobium, Plasmodium falciparum infection and anaemia in children in Accra, Ghana
Source: Trop Dis Travel Med Vaccines. 2018 Apr 18;4:3. doi: 10.1186/s40794-018-0063-7 (PMC5907294; doi:10.1186/s40794-018-0063-7)
Supplement: Supplementary file 1 — Socioeconomic Status Tool. (DOCX 48 kb) [file 40794_2018_63_MOESM1_ESM.docx]

**Additional file 1**

**Socioeconomic Status Tool**

**SOCIO ECONOMIC STATUS (TICK WHICH IS APPROPRIATE)**

**Roof materials ` Water source Occupation of household head**

Grass Piped water/sachet/bottles water none

Plastic sheets Open well Farming

Aluminum sheets Protected well/ Borehole Self employed

Tiles/cement/bricks Lake/pond/ Stream/river Salaried

Piece work

**TOILET FACILITY FLOOR MATERIAL HOUSEHOLD ASSETS**

None Earth/ mud Electricity

WC Cement/concrete/tiles/terrazzo Radio

Pit latrine Wood planks Television/ Phone

KVIP Carpet Refrigerator Bicycle Motor

Car/ truck
